# Clinical significance of preoperative serum interleukin-6 and C-reactive protein level in breast cancer patients

**DOI:** 10.1186/1477-7819-9-18

**Published:** 2011-02-06

**Authors:** Praveen Ravishankaran, R Karunanithi

**Affiliations:** 1 Department of General Surgery, Coimbatore Medical College Hospital, Coimbatore, Tamil Nadu, India; 2 Department of orthopaedics and spine surgery, Ganga Hospital, Coimbatore, Tamil Nadu, India

## Abstract

**Background:**

Breast cancer is a disease that continues to plague females during their entire lifetime. IL-6 and CRP are found to be elevated in various inflammatory and malignant diseases and their levels are found to correlate with the extent of the disease. The primary objective of this study was to determine the preoperative serum levels of IL-6 and CRP in breast carcinoma, and to correlate them with the staging of the disease and the prognosis.

**Methods:**

59 female patients admitted for breast cancer were identified for the study and were subjected to thorough evaluation. Serum levels of IL-6 were assessed via Enzyme-Linked Immuno-Sorbent Assay (ELISA), and CRP was measured via immunoturbidimetry. Histological findings included tumour size, lymph node (LN) metastasis, and tumour staging. Relevant investigations were made to find out the presence of distant metastasis. Statistical analysis of the data was then processed.

**Results:**

Increases in cancer invasion and staging are generally associated with increases in preoperative serum IL-6 levels. IL-6 and CRP levels correlated with LN metastasis (P < 0.001, P < 0.001) and TNM stage (P < 0.001, P < 0.001). Tumour invasion and the presence of distant metastasis is associated with higher IL-6 levels (P = 0.001, P = 0.009). When we established the cutoff value for IL-6 level (20.55 pg/dl) by ROC curve, we noted a significant difference in overall survival (OS; P = 0.008). However, CRP evidenced no significance with regard to patient's OS levels. Serum IL-6 levels were correlated positively with CRP levels (r2 = 0.579, P < 0.01)

**Conclusion:**

Serum levels of IL-6 correlates well with the extent of tumor invasion, LN metastasis, distant metastasis and TNM staging thus enveloping all aspects of breast cancer.

## Introduction

Breast cancer is a disease affecting millions of women as well as men all over the world. The TNM system of classification is used for staging of the disease which has a strong influence on the prognosis of the patient. Wide array of cytokines are secreted by the breast tumours of which IL-6 is one of them. IL-6 is a pleiotrophic cytokine with a wide range functions. IL-6 binds to the IL-6 receptor, activates the Janus kinase (JAK), and subsequently phosphorylates the signal transducers and activators of transcription (STAT). The phosphorylated STAT gene translocates into the nucleus and activates the target genes like VEGF and rho which increases the aggressiveness of the tumour. This involvement of IL-6 at a cellular level with the processes of cancer control is reflected by the results of serum studies of cancer patients, where IL-6 may reflect prognosis and tumour load. Elevated IL-6 levels have been associated with advanced stage and metastasis-related morbidity [1-3]. It has been recently reported that patients with metastatic ovarian cancer and patients with metastatic renal cell carcinoma have higher serum IL-6 levels than those without disseminated disease [4,5]. It has also been demonstrated that elevated IL-6 levels are associated with a poor prognosis in tumours such as non-small-cell lung cancer. The ontological role of IL-6 in this process is not known [6].

C-reactive protein (CRP) is a representative marker for inflammatory conditions, and performs a crucial anti-infection function in the immune system. In many cancers, it has been reported that chronic inflammation is involved with malignant change, and the risks of cancer are increased when pre-diagnostic CRP levels are high [7]. Cancer invasion begins with inflammation around cancer cells. Thus, it has been reported that serum CRP levels are higher in cases of invasive cancer than in cases of non-invasive cancer [8,9].

The principal objective of this study was to determine the relationship between serum IL-6 and CRP levels and staging and prognosis in breast cancer patients.

## Materials and methods

### Patients

Fifty nine cases of breast cancer admitted in our hospital were selected for the study. Basic blood investigations, chest x-ray, ECG and CT scan were done for all the patients and the diagnosis was confirmed. Core needle biopsy was done and the hormone receptor status assessed. Blood samples were drawn for IL-6 and CRP levels on admission.

The patients were then assessed according to the pathological TMN staging

1. Primary tumour (T1 = ≤2 cm, T2 = 2-5 cm, T3 = >5 cm, T4 = chest wall or skin infiltration

2. Nodal staging (N1 = 1-3 nodes, N2 = 4-9 nodes, N3 = > 10 node)

3. Presence (M0) or absence (M1) of metastasis.

The patients were then subjected to surgery with or without neo-adjuvant chemotherapy. Early invasive breast cancer (Stage I, IIa and IIb) was treated with mastectomy and axillary lymph node clearance followed by adjuvant chemotherapy for all node positive breast cancer, all cancers that are larger than 1 cm, tumours with high histological grade and negative hormone receptor status. Advanced locoregional breast cancer (Stage IIIa or IIIb) was treated with modified radical mastectomy followed by adjuvant chemotherapy and radiotherapy if operable and if inoperable neoadjuvant chemotherapy was used to decrease the locoregional cancer burden and permit subsequent surgery. For cases with stage IV breast cancer hormone therapy was done for hormone receptor positive tumours and chemotherapy was given for receptor negative cancers.

Patients were asked to come for follow up once every three months for a duration of two years. Serum levels of IL-6 and CRP were estimated every three months. All patients provided informed consent, and the hospital review board approved the study.

## Assays for serum il-6 and crp

The blood sample for IL-6 collected using standard sampling tubes were transported to the lab within 5 hrs at 20-25°C. The samples for IL-6 were analysed using **Elecsys 2010 cobas e 411 analyser **by Electrochemiluminescence immunoassay. The sandwich principle was used and the total duration of assay took 18 minutes. The measuring range of IL-6 is 1.5-5000 pg/ml(defined by the lower detection limit and the maximum of the master curve). The normal value for IL-6 in a healthy individual is expected to be <7 pg/ml. The samples for CRP were measured immunoturbimetrically using **RANDOX analyser**. Serum is used undiluted and CRP remains stable in the serum for at least 3 days at 15 -25°C. The measuring range of CRP is 0-220 mg/l, the normal value of CRP being <5 mg/l.

## Descriptive statistical analysis

Serum levels of IL-6 and CRP were expressed as the means ± SD. A p value of < 0.05 was considered to be statistically significant and was calculated by one way ANOVA. The Spearman rho correlation coefficient (*r*) was employed to evaluate the correlation between the IL-6 and CRP levels and the clinical findings. The IL-6 and CRP cut-off values for survival analysis were determined by the ROC curve. Survival durations were calculated via the Kaplan-Meier method. Statistical Package for Social Sciences (SPSS) ver. 17 software was used for the statistical analysis.

## Results

### Patient characteristics

The patients were classified by their pathologic characteristics, including tumor size, status of lymph node metastasis, presence or absence of metastasis and TNM staging. The patients consisted of 59 women, with a median age of 59.11 years (range, 36-85 years). In all 5 patients had stage 2A disease(8.4%), 8 patients belong to stage 2B(13.6%) 15 patients belong to stage 3A(25.42%),13 belongs to stage 3B(22.03%) 10 patients to stage 3C(16.94%) and 8 to stage 4(13.56%). The other patient characteristics are summarized in Table 1.

**Table 1:** Patient characteristics

	No. of patients	%
Total number of patients	59	

Age		

Median(Range)	59.11(36-85)	

Depth of tumor invasion		

pT1	7	11.9

pT2	15	25.4

pT3	14	23.7

pT4	23	38.9

LN metastasis		

N0	5	8.6

N1	17	28.8

N2	21	35.6

N3	16	27.1

Distant metastasis		

Metastasis(-)	51	86.4

Metastasis(+)	8	13.6

The relationships between IL-6, CRP levels, and clinico-pathologic variables are provided by the Spearman rho correlation coefficient (*r*) in Table 2.

**Table 2:** Correlation between the IL-6, CRP and clinicopathological parameters

	IL-6			CRP		
	**Median ± SD**	***r***	***P***	**Median ± SD**	***R***	***P***

**Total**	**pg/ml**			**(mg/dl)**		

**Tumor depth**						

pT1	8.1 ± 5.7	0.564	0.001	8.4 ± 3.1	0.374	0.304

pT2	17.8 ± 8.6			10.5 ± 2.7		

pT3	19.2 ± 10.0			9.0 ± 2.4		

pT4	26.2 ± 11.2			16.0 ± 3.3		

**LN meta**						

N1	11.6 ± 4.8	0.844	0.000	9.6 ± 4.1	0.690	0.000

N2	20.7 ± 6.9			15.7 ± 9.0		

N3	32.1 ± 10.7			29.4 ± 15.6		

**Distant meta**						

Metastasis(-)	17.3 ±7.6	0.773	0.009	13.9 ± 8.5	0.175	0.061

Metastasis(+)	39.7 ± 9.3			37.4 ± 16.0		

**TNM stage**						

2A	5.6 ± 1.5	0.702	0.000	10.1 ± 3.9	0.463	0.000

2B	11.7 ± 4.4			9.2 ± 4.6		

3A	16.9 ± 4.7			13.8 ± 7.2		

3B	19.1 ± 4.8			12.8 ± 9.2		

3C	26.3 ± 7.0			21.5 ± 9.9		

4	39.8 ± 9.4			37.5 ± 16.0		

### Clinicopathological significance of IL-6 in breast cancer

**Figure 1:**
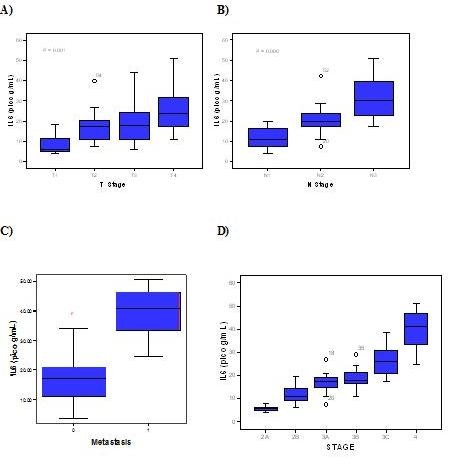
**IL-6 and the characteristics of breast tumour.** (A) IL-6 levels according to tumor depth. (B) IL-6 levels according to LN metastasis. (C) IL-6 levels according to the metastasis. (D) IL-6 levels according to TNM staging.

We noted that IL-6 levels were significantly correlated with the tumour size with higher IL-6 levels was detected in tumours sized ≥ 5 cm (*P *= 0.001, r = 0.564). Additionally, with increasing degrees of tumour invasion, the median levels of IL-6 evidenced a tendency to increase, and this difference in IL-6 levels was found to be statistically significant (*P *< 0.001). In cases of LN metastasis, we also noted a significant difference between the serum level of IL-6 and LN metastasis (*P *< 0.001, r = 0.844). The median level of IL-6 increased proportionally with the stage of the cancer (the median level of IL-6 in stage 2a 5.6 ± 1.5 pg/ml, stage 2b 11.7 ± 4.4 pg/ml, stage 3a 16.9 ± 4.7 pg/ml, stage 3b 19.1 ± 4.8 pg/ml, stage 3c 26.3 ±7.0 pg/ml and stage 4 39.8 ±9.4 pg/ml), and this difference was statistically significant (*P *< 0.001). Additionally, serum IL-6 levels were significantly higher in patients with distant metastasis (39.7 ±9.3 pg/ml) than in those without distant metastasis(17.3 ± 7.6 pg/ml) whose difference is also statistically significant(P < 0.001) (Figure 1).

(A) IL-6 levels according to tumor depth. (B) IL-6 levels according to LN metastasis. (C) IL-6 levels according to the metastasis. (D) IL-6 levels according to TNM staging.

The patients were divided into two groups on the basis of an IL-6 cutoff value of 20.3 pg/ml by the ROC curve with a sensitivity of 88.6% and a specificity of 54.1%.

**Figure 2:**
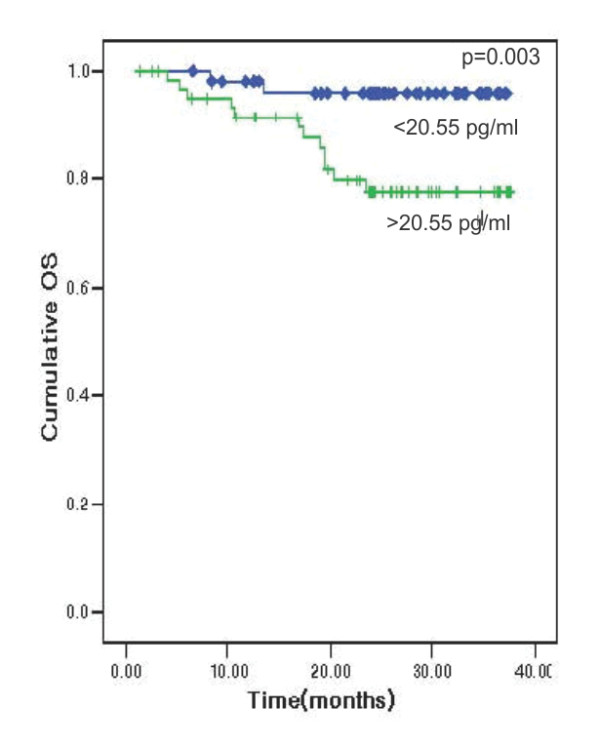
**Overall survival curve according to IL-6 (Interleukin-6) levels**.

We noted significant differences in the Overall Survival between the two groups (82.7% versus 97.2%; *P *= 0.008) See Figure 2 for Overall survival curve according to IL-6 (Interleukin-6) levels.

### Clinicopathological significance of CRP

**Figure 3:**
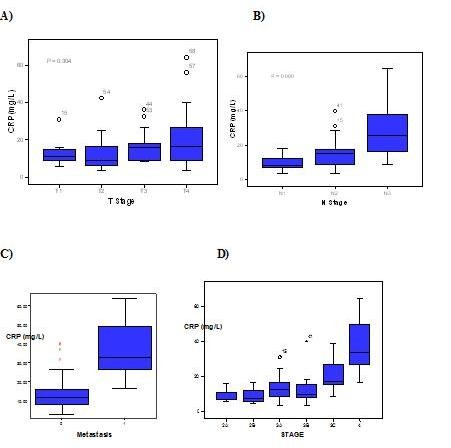
**CRP and the characteristics of breast tumour.** (A) CRP levels according to tumor depth. (B) CRP levels according to LN metastasis. (C) CRP levels according to distant metastasis. (D) CRP levels according to TNM staging.

We noted that CRP levels did not differ significantly with tumour size (r = 0.374, *P *= 0.304). However we noted significant differences in serum CRP levels between patients with lymph node metastasis and those without lymph node metastasis (r = 0.690, *P *= 0.000). The median levels of CRP increased with increasing stage, and we also noted significant differences between the CRP level and cancer stage (the median level of CRP in stage 2a 10.1 ± 3.9 mg/dl, stage 2b 9.2 ± 4.6 mg/dl, stage 3a 13.8 ± 7.2 mg/dl, stage 3b 12.8 ± 9.2 mg/dl, stage 3c 21.5 ± 9.9 mg/dl and stage 4 37.5 ± 16.0; *P *< 0.001). The CRP levels did not differ significantly in patients with metastasis (37.4 ± 16.0 mg/dl) as compared to those without metastasis (13.9 ± 8.5 mg/dl, *P *= 0.061). Figure 3 shows (A) CRP levels according to tumor depth. (B) CRP levels according to LN metastasis. (C) CRP levels according to distant metastasis. (D) CRP levels according to TNM staging.

15.5 mg/dl was taken as the cutoff value of CRP by ROC curve, after which the patients were divided into two groups. The sensitivity and specificity of 15.5 mg/dl as the cutoff value were 62.1% and 75.3% on OS. We noted no significant difference in the OS values (84.4% vs 92.3%, *P *= 0.197) among the groups.

### Association between IL-6 and CRP

**Figure 4:**
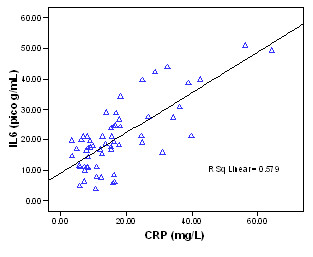
**Correlation between IL-6 and CRP in breast cancer**.

Serum IL-6 levels also correlated positively with that of CRP (r^2 ^= 0.579, p < 0.01) thus proving a positive association between the two variable (Figure 4).

## Discussion

It has been long established that the pathologic variables of tumour size, lymph node status, and histologic tumour grade are significant prognostic indicators in breast carcinoma [10-13]. More recently, biomarkers of prognosis have been identified [14-16] and a radiological predictor of survival has been discovered [17], but the value of tumour size, lymph node status, and tumour grade as powerful predictors of survival remains [18].

In this study, the serum levels of both IL-6 and CRP evidenced statistically significant differences related to the stage of LN metastasis. The serum levels of IL-6 evidenced statistically significant differences with relation to changes in tumor size. As the stage of the disease increased, serum IL-6 and CRP levels increased proportionately. Additionally, the median levels of IL-6 were significantly higher in the patients with distant metastasis than in those without distant metastasis, but in CRP, this was not proven. We also noted a significant association between the levels of IL-6 and CRP (p < 0.01).

Thus the levels of IL-6 correlates with all the aspects of breast cancer like tumour size lymph node involvement, distant metastasis and the final TNM staging of the disease. The overall survival of the patient also seems to be affected in patients with elevated levels of IL-6. The levels of CRP correlated only with lymph node metastasis and not with tumour size and distant metastasis. CRP also does not correlate with the overall survival of the patient.

It has been proved that TNM staging correlates with the prognosis of patients with breast cancer. As IL-6 has a direct correlation with the stage of the disease it may indirectly correlate with the prognosis of the patient unlike that of CRP.

Interleukin-6 (IL-6) is a multi-poietic cytokine that induces the growth and differentiation of immune cells, the production and expression of other cytokines, and acute-phase protein synthesis. IL-6 also exerts several effects on cancer cells[19,20]. In the development and progression of cancer, angiogenesis is a crucial and essential process. IL-6 is associated with angiogenesis by virtue of its ability to induce the mRNA of vascular endothelial growth factor (VEGF), which is typically a direct angiogen [19]. Additionally, IL-6 activates the Rho protein, which is associated with cell-cell adhesion and invasion in malignancy [21]. Together these factors increase the aggressiveness of the tumour. It has been indicated in this study that IL-6 level increases as the aggressive behaviour of the tumour increases (IL-6 levels increase as the stage of the cancer increases).

CRP is generated by the liver and other organs in response to the release of IL-6 by monocytes and other immune cells. Thus, when IL-6 levels increased, CRP levels also increased. This has been proven by the positive association between IL-6 and CRP in this study.

## Conclusion

Thus the levels of IL-6 has a positive correlation with TNM staging system of breast cancer thus indirectly correlating with the prognosis of the patient. CRP estimation does not seem to be very useful in evaluating the patient with breast cancer, though its level correlates with that of IL-6.

## Limitations of the study

1) A larger sample size needs to be evaluated to reach a definite conclusion.

2) A longer follow up of the patient is also essential for completeness and is currently underway.

## Authors' contributions

PR conceived the study, collected the data and drafted the manuscript. KR participated in the design of the study and performed the statistical analysis. Both the authors read and approved the final manuscript.
